# Prise maternelle d’anti-inflammatoires non stéroïdiens et fermeture du canal artériel

**DOI:** 10.11604/pamj.2016.25.251.10317

**Published:** 2016-12-21

**Authors:** Daouda Diamane Ndour

**Affiliations:** 1 Service de Néonatologie et Réanimation Néonatale, Centre Hospitalier Universitaire Albert Royer Dakar, Université Cheikh Anta Diop de Dakar BP: 5005 Dakar-Fann, Sénégal

**Keywords:** Fermeture prématurée in utero du canal artériel, AINS, anasarque fœtal, insuffisance cardiaque, Intrauterine premature closure of the ductus arteriosus, NSAID, fetal anasarca, heart failure

## Abstract

Nous rapportons un cas de fermeture prématurée in utero du canal artériel diagnostiqué chez un fœtus en état d’anasarque avec insuffisance cardiaque majeure après prise d’anti-inflammatoire non stéroïdiens (AINS) par la mère. Une échographie obstétricale au deuxième trimestre a permis la découverte d’une asymétrie des cavités cardiaques. L’indication de césarienne a été posée à 30 semaines d’aménorrhées devant un fœtus en état d’anasarque avec insuffisance cardiaque majeure sur une fermeture anténatale du canal artériel. A partir de ce cas nous nous proposons de réaliser une revue de la littérature pour illustrer les complications fœtales et néonatales potentielles résultant de la fermeture précoce du canal artériel secondaire à la prise d’AINS pendant la grossesse.

## Abstract (en)

We report a case of intrauterine premature closure of the ductus arteriosus diagnosed in an anasarca fetus associated with major heart failure following mother’s ingestion of non-steroidal anti-inflammatory drugs (NSAID). Second trimester obstetric ultrasound allowed detection of an asymmetry of the cardiac chambers. Caesarean section was perfomed at 30 weeks of pregnancy to remove anasarca fetus with major heart failure due to prenatal closure of the ductus arteriosus. Based upon this case, we conducted a literature review to highlight potential fetal and neonatal complications resulting from early closure of the ductus arteriosus secondary to NSAID use during pregnancy.

## Introduction

Le canal artériel est un vaisseau sanguin qui dérive du sixième arc aortique gauche distal. Il est situé entre l’artère pulmonaire et l’aorte descendante, réalisant un shunt partiel de la circulation pulmonaire en constituant la voie d’éjection principale du ventricule droit dès 6 semaines d’aménorrhées. La circulation sanguine fœtale est une circulation en parallèle avec 2 shunts: le Foramen ovale: le sang oxygéné venant de la veine ombilicale est préférentiellement dirigé vers le ventricule gauche via le foramen ovale, ainsi la circulation cérébrale et coronaire reçoit du sang plus oxygéné; le canal artériel permet au débit du cœur droit provenant essentiellement de la veine cave supérieure d’aller vers l’aorte et la circulation placentaire. Les poumons étant non fonctionnels seulement 10% du débit sanguin fœtal se dirige vers la circulation pulmonaire du fait des résistances vasculaires pulmonaires élevées.

Pendant la grossesse, le maintien de l’ouverture du canal artériel est essentiel et est sous la dépendance d’agents vasodilatateurs. Ils visent à contre-balancer les mécanismes naturels pro contractiles représentés par le tonus intrinsèque des fibres musculaires lisses ductales soumis aux différents neurotransmetteurs, il s’agit donc d’un phénomène de vasodilatation active. Les prostaglandines, plus particulièrement la PGE2 et la PGI2 sont probablement les agents vasodilatateurs les plus importants pour le maintien de l’ouverture du canal artériel en deuxième partie de grossesse.

A la naissance, l’exclusion de la circulation placentaire et l’aération pulmonaire (entraînant une augmentation de la saturation en oxygène du sang) aboutissent à la fermeture du canal artériel. La fermeture normale du canal artériel apparaît donc dans les premiers jours après la naissance. Elle s’effectue en plusieurs étapes: la contraction initiale des fibres musculaires de la paroi ductale entraîne une oblitération fonctionnelle de la lumière avec une diminution de la sensibilité des fibres musculaires aux agents vasodilatateurs qui va prévenir sa réouverture, enfin des remaniements histologiques sont responsables de la fermeture anatomique définitive du canal artériel. Nous rapportons un cas de fermeture prématurée in utero du canal artériel diagnostiqué chez un fœtus en état d’anasarque avec insuffisance cardiaque majeure après prise d’anti-inflammatoire non stéroïdiens (AINS) par la mère.

## Patient et observation

Une deuxième part âgée de 34 ans est adressée au pédiatre cardiologue pour une découverte à l’échographie obstétricale du deuxième trimestre d’une asymétrie des cavités cardiaques fœtales. La dame, sans antécédents particuliers rapporte sans précisions une prise de médicaments AINS durant la grossesse. La grossesse se déroulait jusque-là sans particularités avec une bonne mobilité et une bonne croissance fœtales. L’échographie cardiaque confirme la disparité nette des cavités cardiaques aux dépens du ventricule gauche. Cependant il n’y a pas d’obstacle sur la valve pulmonaire et le canal artériel est constrictif. Le gradient de pression dans le canal artériel de 28 mm Hg et la vitesse 2,5m/s (valeur très élevée en anténatale) Il s’agit donc d’une grossesse de 28 semaines d’aménorrhées (SA) avec un tableau de fermeture progressive in utéro du canal artériel, sans épanchement liquidien.

En accord avec les obstétriciens nous décidons d’une poursuite de la grossesse avec une surveillance rapprochée. L’échographie à 30 SA retrouve un ventricule droit dilaté, hyper échogène et hypokinétique. Le canal artériel n’est pas visible, il y a une fuite tricuspide et la fréquence cardiaque est normale. L’échographie morphologique retrouve une ascite et un aspect d’anasarque fœtal. Il s’agit donc d’une grossesse de 30 SA avec tableau d’insuffisance cardiaque majeure et anasarque fœtal sur une fermeture anténatale du canal artériel. L’extraction est alors réalisée en accord avec les parents. L’échographie post natale confirme la dilatation des cavités droites et l’absence de canal artériel. Le séjour est soins intensifs néonatal est classique et se passe finalement sans particularités.

## Discussion

Les anti-inflammatoires non stéroïdiens sont couramment utilisés et agissent en inhibant la synthèse des prostaglandines par inhibition de la cyclo-oxygénase. Ce mécanisme d´action peut conduire ainsi à la fermeture du canal artériel. Il est fortement déconseillé aux femmes enceintes d´utiliser tout AINS, en particulier dans le dernier trimestre de la grossesse, en raison des risques fœtaux. La fermeture en période anténatale du canal artériel est possible. Elle peut être spontanée ou secondaire à l´utilisation d´inhibiteurs de prostaglandine-synthétase tels que les AINS et les glucocorticoïdes [1, 2].

In utero le canal artériel est crucial pour le maintien de la circulation fœtale. Le ventricule droit du fœtus éjecte 60 à 65% du débit cardiaque totale. L’essentiel du débit d’éjection du ventricule droit (75%) passe dans l´aorte grâce au canal artériel, tandis que seulement 25% perfuse le lit vasculaire pulmonaire du fait des résistances élevées [3]. La fermeture in utero du canal artériel provoque l’éjection dans la circulation pulmonaire de tout le débit du ventricule droit, conduisant à une augmentation importante des pressions dans les artères pulmonaires, l´hypertrophie des médias et l´hypertension pulmonaire. L’augmentation de la post charge du ventricule droit peut conduire à une hypertrophie, un dysfonctionnement et une insuffisance tricuspide [4, 5]. En outre, le dysfonctionnement du cœur droit peut conduire à une insuffisance cardiaque congestive fœtale et un état anasarque.

Dans notre cas la fermeture prématurée du canal artériel est probablement due à la prise d’ibuprofène par la mère. Aravind T. Shastri et al [6] rapportent un cas de fermeture prématurée in utero du canal artériel diagnostiqué en postnatale chez un enfant présentant une insuffisance cardiaque et un état d’anasarque. Une échographie cardiaque réalisée 6h après la naissance a montré une dilatation marquée de l´oreillette droite, un ventricule droit hypertrophié, des pressions pulmonaires élevées, un petit épanchement péricardique, et un canal artériel fermé. La mère ignorait sa grossesse jusqu´à ce qu´elle entre dans la phase du travail et elle avait pris des comprimés de diclofénac dans les mois précédents.

Dans la littérature certains auteurs rapportent des cas de fermeture prématurée du canal artériel [7, 8] qui étaient associé à la prise maternelle d´aspirine ou d’indométacine. Pour d’autres auteurs la fermeture prématurée du canal artériel étaient sans rapport avec une prise maternelle de médicament. Mielke et al. [9] ont ainsi décrit un cas de dilatation des cavités droites avec une insuffisance tricuspide sévère en raison d’une constriction du canal artériel; les symptômes ont disparu en post partum à 3 jours de vie. Trevett et al. [10] ont présenté le cas d´un fœtus de 33 semaines avec hypertrophie du ventricule droit secondaire à une constriction du canal artériel. Le fœtus a été surveillée par échographie hebdomadaire pour des signes d´anasarque, puis le travail a été déclenché à 38 semaines; l’enfant est né asymptomatique, mais à 3 semaines de vie l’échographie cardiaque retrouvait une hypertrophie ventriculaire droite modérée et une hypertrophie ventriculaire gauche minimale.

Le but de cet article est de rappeler aux cliniciens les complications cardiaques possibles fœtales et néonatales résultant de l´exposition in utero aux médicaments AINS. La fermeture prématurée du canal artériel est une des hypothèses à examiner devant des signes de dilatation des cavités droite ou d’insuffisance cardiaque globale en l´absence d´une anomalie cardiaque structurelle [5], ainsi que chez tout nouveau-né présentant un état d’anasarque inexpliquée. Il nous semble également intéressant d’examiner minutieusement la liste de tous les médicaments ingérés par la mère en anténatal.

## Conclusion

La fermeture anténatale du canal artériel est possible. Elle peut être spontanée ou provoquée par la prise d’anti inflammatoire au cours de la grossesse. Elle se manifeste par un retentissement sur la circulation droite et nécessite une surveillance étroite. L’indication d’extraction se pose en cas décompensation cardiaque en ne sous-estimant pas les conséquences de la prématurité induite. L’intérêt de ce cas clinique repose sur le fait de souligner qu’il faut savoir rechercher une fermeture prématurée du canal artériel en cas d’épanchements inexpliqués…
